# Application of the robot-assisted implantation in deep brain stimulation

**DOI:** 10.3389/fnbot.2022.996685

**Published:** 2022-12-02

**Authors:** Fang-Zhou Ma, De-Feng Liu, An-Chao Yang, Kai Zhang, Fan-Gang Meng, Jian-Guo Zhang, Huan-Guang Liu

**Affiliations:** ^1^Department of Neurosurgery, Beijing Tiantan Hospital, Capital Medical University, Beijing, China; ^2^Department of Functional Neurosurgery, Beijing Neurosurgical Institute, Capital Medical University, Beijing, China; ^3^Beijing Key Laboratory of Neurostimulation, Beijing, China

**Keywords:** accuracy, deep brain stimulation, robot-assisted implantation, videometric tracker, safe

## Abstract

**Introduction:**

This work aims to assess the accuracy of robotic assistance guided by a videometric tracker in deep brain stimulation (DBS).

**Methods:**

We retrospectively reviewed a total of 30 DBS electrode implantations, assisted by the Remebot robotic system, with a novel frameless videometric registration workflow. Then we selected 30 PD patients who used stereotactic frame surgery to implant electrodes during the same period. For each electrode, accuracy was assessed using radial and axial error.

**Results:**

The average radial error of the robot-assisted electrode implantation was 1.28 ± 0.36 mm, and the average axial error was 1.20 ± 0.40 mm. No deaths or associated hemorrhages, infections or poor incision healing occurred.

**Conclusion:**

Robot-assisted implantation guided by a videometric tracker is accurate and safe.

## Introduction

Kwoh et al. (1988) firstly used the position information obtained from images by PUMA206 robot to perform stereotactic biopsy surgery. This is the first clinical application of neurosurgical robot. Neurosurgical robot is mainly used for biopsy, deep brain stimulation, stereoelectroencephalogram, hematoma aspiration and other operations (Ahmed et al., 2018). Robot control technology and friendly man-machine interface technology, greatly improve the precision and dexterity of surgery (can eliminate the tremor of the hand, improve the skill of the doctor). The combination of machine and human intelligence gives full play to the advantages of human in thinking and logical reasoning, learning and skill growth, experience and rapid decision making, combined with the machine's strong ability in repetition and consistency, fatigue resistance, continuous operation, so that the two forms complementary advantages. Video tracking and positioning is an advantage of robots that can be used in neurosurgery for precise electrode implantation.

Deep brain stimulation (DBS) is a powerful method in the management of Parkinson's disease. Currently, there are two most commonly used DBS targets for Parkinson's disease (PD): STN and GPi (Deuschl et al., 2006; Carmona-Torre et al., 2013; Rowland et al., 2017). After stimulation of STN in PD patients, the dosage of levodopa was reduced. However, the cognitive function of PD patients may be slightly lower than that before surgery. Using GPi as a therapeutic target is relatively less cognitively and spiritually damaging. In addition to STN and GPi, PPN was targeted for DBS treatment for gait symptoms of PD improved (Collomb-Clerc and Welter, 2015). In addition, previous studies have shown that the zona incerta (ZI) as a target has unique therapeutic advantages in PD, ET and dystonia (Burrows et al., 2012; Falconer et al., 2018; Holslag et al., 2018). However, the procedure is associated with adverse effects, mainly neurocognitive, and with side-effects created by spread of stimulation to surrounding structures, depending on the precise location of electrodes (Benabid et al., 2009).

DBS electrode implantation can be achieved in a variety of ways, with the 2 main categories including frameless and frame-based systems (Vadera et al., 2017). Traditionally, neurosurgeons have used frame-based stereotaxy, microelectrode recordings (MERs) and awake macrostimulation testing to place DBS leads and verify optimal placement (Amirnovin et al., 2006; Starr et al., 2006). Frame-based systems are considered to be the “gold standard” (Hemm and Wårdell, 2010). Whereas, robots can effectively position, orient and manipulate surgical tools in 3D space with a high level of accuracy (Li et al., 2002), some commercially available frameless robotic systems, such as NeuroMate, ROSA and Renaissance (Faria et al., 2015), have been used in DBS and have provided accuracy comparable to the frame-based procedure (Varma and Eldridge, 2006; Goia et al., 2019; VanSickle et al., 2019). Most recently, robot-assisted techniques are gaining traction for DBS, with adoption in Europe, Asia, and the United States (Lefranc and Le Gars, 2012; Lin et al., 2019; Liu et al., 2020). The objective of this study is to demonstrate the validity of robot-assisted DBS procedures guided by a videometric tracker (Schneider and Feussner, 2017). This study used the Remebot robotic system (Beijing Baihui Weikang Technology Co., Ltd.; Beijing, China), which gained CFDA clearance for neurosurgery in 2018. It is an image-guided, computer-controlled, videometric-tracking-guided robotic system and unique in utilizing the optical tracking to achieve registration and navigation in DBS procedures (VanSickle et al., 2019; Varma and Eldridge, 2006; Goia et al., 2019).

## Patients and methods

We retrospectively reviewed a total of 30 patients with Parkinson's disease qualified for a DBS procedure (Table 1). All underwent DBS electrode implantation assisted by the Remebot robotic system. Of the 30 patients, 17 were male, and 13 were female. The mean age at the time of surgery was 56.7 ± 9.2 (45–70) years, with a mean disease duration of 8.2 ± 3.3 (5–15) years. Then we selected 30 PD patients who used stereotactic frame surgery to implant electrodes during the same period, and implanted a total of 60 electrodes. The mean age at the time of surgery was 57.3 ± 5.2 (45–70), with a mean disease duration of 8.4 ± 3.6 (5–16) years. There were no statistical differences in age and course of disease between the two groups. All surgeries were performed by the same surgeons from the Department of Neurosurgery at Beijing Tiantan Hospital between October 2018 and June 2021. This study was approved by the Ethics Committee of Beijing Tiantan Hospital (Grant No. QX201600-706) and all patients or their relatives signed informed consent documents.

**Table 1:** Population characteristics.

	**Robot assisted surgery**	**Conventional surgery**	***P* score**
Patients (no.)	30	30	
Age (yrs)	56.7 ± 9.2 (45–70)	57.3 ± 5.2 (45–70)	*P* > 0.05
Sex			
Male	17	20	
Female	13	10	
Disease duration (yrs)	8.2 ± 3.3 (5–15)	8.4 ± 3.6 (5–16)	*P* > 0.05
Implanted electrodes (no.)	60	60	

### Surgical procedure

One to 2 days prior to surgery, all patients underwent a magnetic resonance imaging (MRI) (3.0 Tesla, Siemens, Germany). To guarantee the visualization of the anatomical structures of interest, T1WI-3D-MPRAGEMR imaging (slice thickness 1.0 mm, TR 6.4 ms, TE 3.0 ms, interslice gap 0 mm, flip angle 8°), axial and coronal volumetric T2-weighted MR imaging (slice thickness 2.0 mm, TR 3000 ms, TE 130 ms), sagittal, axial and coronal fluid attenuated inversion recovery (slice thickness 1.0 mm, TR 4800 ms, TE 228.2 ms) were performed. The images were all fused together to plan the targets and trajectories by Neurosurgical navigation software (RemebotSPS, Beijing Baihui Weikang Technology Co., Ltd., China).

**Figure 1:**
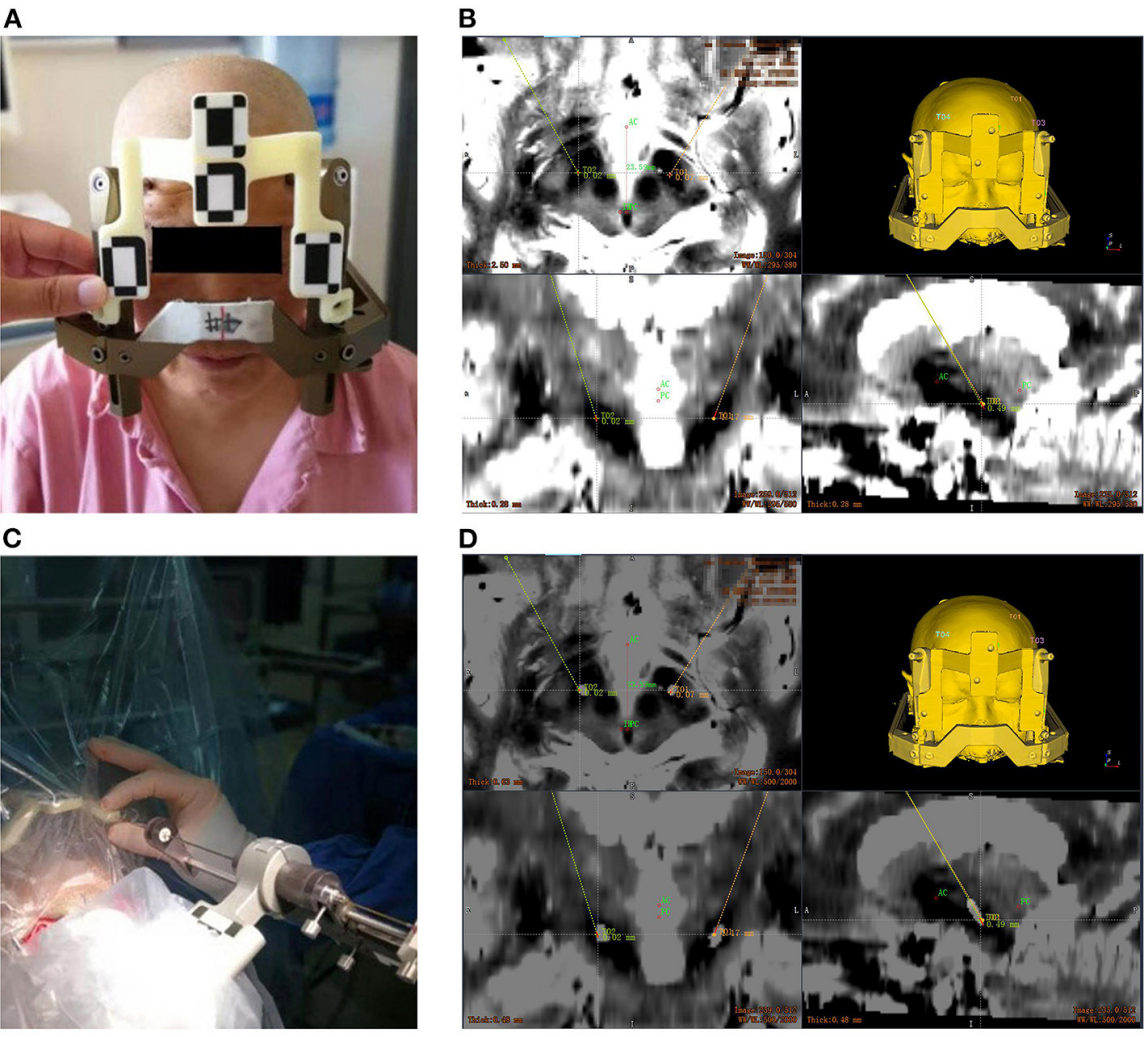
Electrode implantation in a DBS procedure assisted by the Remebot robotic system. **(A)** The optical marker screwed to the Leksell frame; **(B)** the target points and trajectories planned upon the fusion of preoperative MR and CT images; **(C)** the verification of accuracy after the automatic optical registration; **(D)** overlay of the postoperative CT images and preoperative MR images with bilateral target trajectories.

On the day of surgery, the Leksell frame was fixed to the patient's head to keep the patient's head in place throughout the DBS procedure, rather than being used as a stereotactic localizer. As shown in Figure 1 the optical frame marker, was capable of automatic patient-to-image registration, and screwed to the Leksell frame without extra injuries. Following this, an axial volumetric computed tomography (CT) (slice thickness 0.625 mm, interslice gap 0 mm, 120 kVp) scan was taken. Due to MRI distortions (Benabid et al., 2009; Guo et al., 2018), MRI image and CT image are fused in the Remebot software as a reference examination. The surgical navigation software realizes the fusion of CT and MRI through mutual information registration algorithm. The accuracy of fusion is judged by doctors, mainly based on the alignment of the same physiological structure information of the two registered images, such as cerebral arterial circle, ventricular horn, temporal pole, etc. The surgical planning was performed after segmenting the intracranial tissue of interest. For skin and bone, marching cubes algorithm was used to achieve 3D structure extraction; for cerebral cortex, brain region segmentation was achieved by standard brain template matching, and then structure extraction was achieved by volume rendering algorithm. The trajectory avoided blood vessels and ventricles during surgical planning.

**Figure 2:**
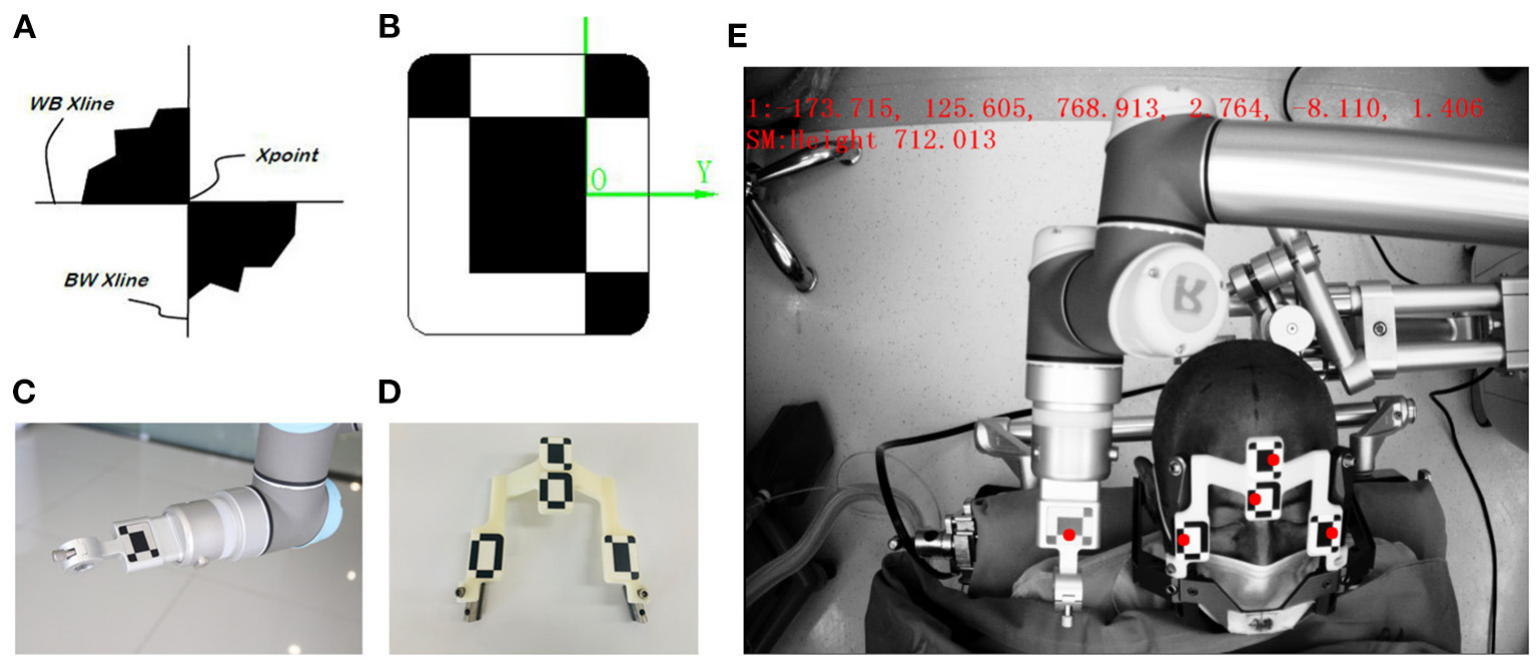
The videometric-tracked target pattern. **(A)** X point; **(B)** a target pattern, consisting of X points geometrically defined and arranged according to specific rules, and its local coordinate system; **(C)** the target pattern engraved on the end effector attached to the robotic arm; **(D)** the target patterns on the frame marker with fiducial balls as close as possible to each origin; **(E)** the camera recognizes the locations of target patterns tracked in the field of measurement (FOM).

The videometric tracker integrated by the Remebot robotic system is a commercially available third-generation stereoscopic optical tracking product (Sánchez-Margallo et al., 2014; Choi et al., 2019). The MicronTracker (ClaroNav, Canada) has been used in rigid tissue surgical navigation, such as orthopedic surgery (Wang et al., 2018) [other than for neurosurgery (Wei et al., 2011)], maxillary orthognathic surgery (Choi et al., 2019) and laparoscopic surgery (Sánchez-Margallo et al., 2014). Tracked objects are marked with a visible target pattern which consists of high-contrast black-white interleaved regions called X points (Figure 2). All target patterns are unique. The tracker's calibration accuracy is 0.2 mm, absolutely matching that of other optical tracking systems (Eggers et al., 2006; Batista et al., 2019; Choi et al., 2019).

Optical frame markers generally consist of intersections as shown in Figure 2A. The optical frame marker consisted of target patterns comprising checkered target regions referred to as “X Point” that can be pinpointed on video image sequences and fiducial balls. The three intersection points are located in the same plane, which can form a coordinate system. The midpoint of the long axis of the intersection point is the origin of coordinates, the direction of the long axis is the X axis, the direction perpendicular to the plane is the Z axis, and the Y axis is determined according to the right-handed coordinate system. Because the relative positions of the three intersections are fixed, each designed Marker is uniquely determined according to geometric relations. In this way, Marker can be used as an object that can be independently tracked and located by binocular camera system. Marker is used for positioning of binocular camera system. By setting the marker at the position of the manipulator and the patient's head, the binocular camera system can track the position of the manipulator and the patient in real time. The camera system on the robot adopts the structure of binocular camera, imitates the principle of human eye imaging, relies on visible light, uses triangulation and edge detection technology to identify the black and white intersection points in Marker, and accurately calculates the three-dimensional coordinates of the intersection points in the camera system's own coordinate system, and then realizes the coordinate positioning of space points.

**Figure 3:**
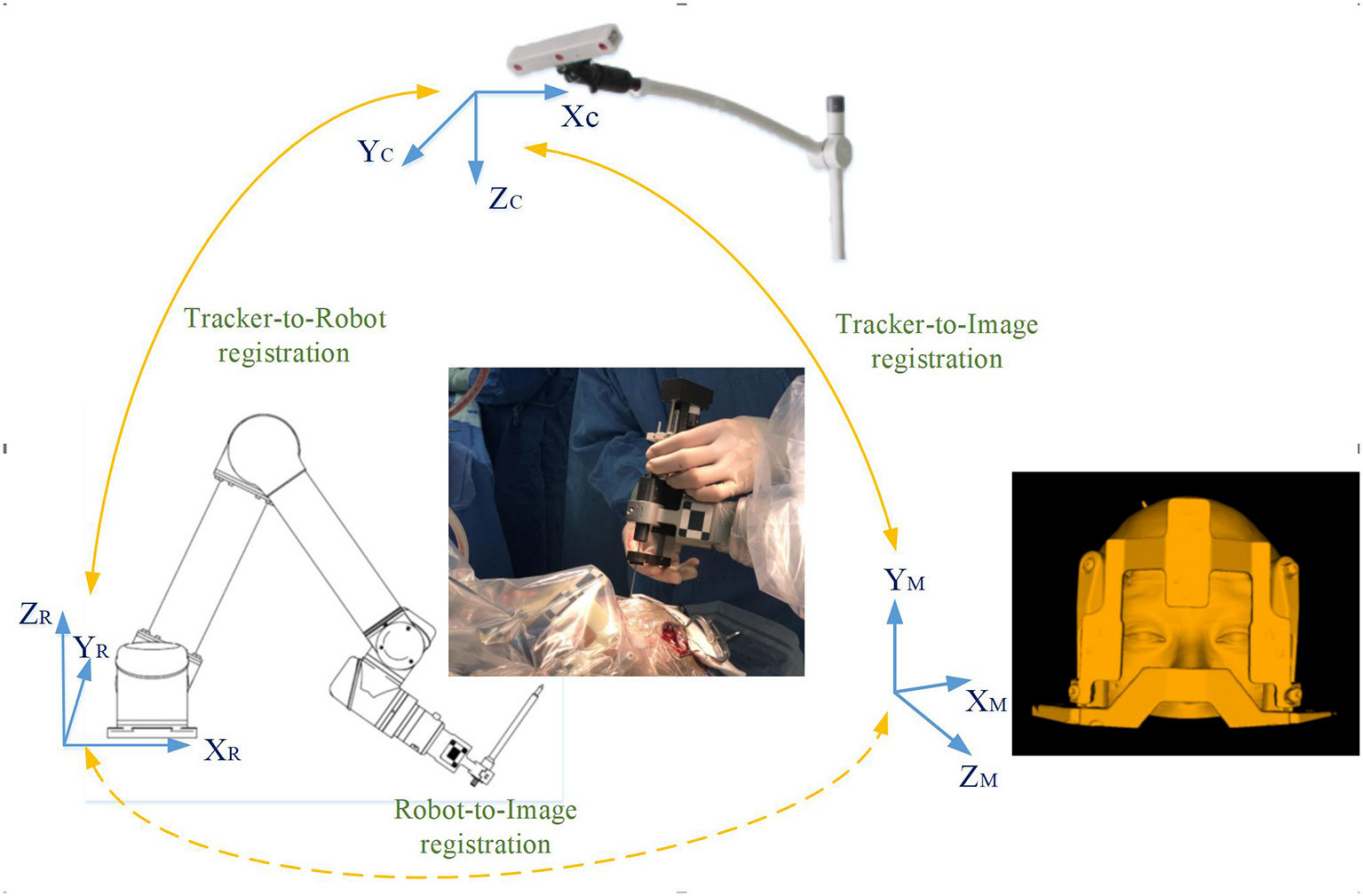
Registration workflow of the correlation between different spaces: (1) tracker-to-image registration: The tracker-to-image registration was achieved by pairing the centers of 4 ceramic balls on the optical frame marker as fiducial points from the image space and the tracker space; (2) tracker-to-robot registration: In the same way, the optical frame marker on the robotic arm is confirmed; (3) robot-to-image registration: the robotic arm automatically moved to certain poses surrounding the patient's head, and the coordinates of that fiducial point in separate spaces were automatically obtained from the robot forward calculation and the tracker. Then the robot-to-image registration was accomplished.

In the operating room, the patient was placed in supine position, the robot operating platform was fixed on the left side of the patient, the Leksell frame was fixed on the operating table, and the head frame and platform were rigidly fixed by the mechanical support arm. The videometric tracker (MicronTracker, ClaroNav, Canada) is equipped with 3 stereo cameras supported by a separate stand mounted above the patient's head to detect optical frame markers within the tracker's field of measurement (FOM). Then, different spaces were associated, which mainly consists of two steps: (1) tracker-to-image registration and (2) tracker-to-robot registration, as shown in Figure 3. The tracker-to-image registration was achieved by pairing the centers of 4 ceramic balls on the optical frame marker as fiducial points from the image space and the tracker space. And then a fiducial point was marked automatically by calculating the center of a series of high-contrast circular zones on the view of preoperative CT. In the tracker space, fiducial points were automatically obtained from the tracker based on specific transformations between the centers and the X points. The specific registration process has been described in detail in our previous study (Liu et al., 2021a,b). Briefly, the registration error was validated < 0.3 mm in the tracker-to-image registration and < 0.08 mm in the tracker-to-robot registration. Then, the robot and the image were registered through the above association, and the data was transferred. The of videometric registration takes about 5–10 min.

Surgical procedures have also been described in previous studies by our group (Liu et al., 2021a,b,c).

Briefly, the robotic arm automatically marked the scalp entry points. After anesthesia, bilateral scalp incisions and drilling were performed under the guidance of a robotic arm. Damage to the dura was avoided as much as possible to prevent premature cerebrospinal fluid loss and subsequent brain shift. Then, the robotic arm was oriented to the trajectory with a micro-drive device. Electrocoagulation needle punctured the dura, hemostasis was achieved and the cannula advanced along the trajectory. Following this, microelectrode recording (MER) was performed. One microelectrode was iteratively advanced in millimeter steps along the planned trajectory until it was positioned 5 mm from the target, and then half a millimeter between iterations. During each step, the neuroelectrical signals coming from neurons were recorded to map the sensorimotor region. After verifying the optimal placement within the target structure based on the recorded data, the microelectrode was replaced with a DBS stimulation electrode, which was also used to perform intraoperative stimulation. With increasing stimulation signal properties, we qualitatively evaluated the patient's symptoms, seeking an optimal clinical outcome with minimal side effects. The electrodes were fixed to the skull when the physiological and clinical criteria for successful placement were fulfilled (Starr et al., 2010). The entire intraoperative process was repeated for the other side.

In the conventional surgery group, patients were also scanned with MRI and CT (installation of the orientation instrument frame), the specific parameters are the same as before. The targets and trajectory of the electrode implantation were calculated and determined using the Leksell SurgiPlan station (Elekta). Then, the anterior (AC) and posterior (PC) commissures were located in the surgical planning workstation, and locate the midpoint of the AC-PC line as the brain origin. Then the spatial coordinates of the target point were determined (Starr, 2002). The subsequent electrophysiological positioning process is roughly the same between the two groups.

### Electrode accuracy and complications

**Figure 4:**
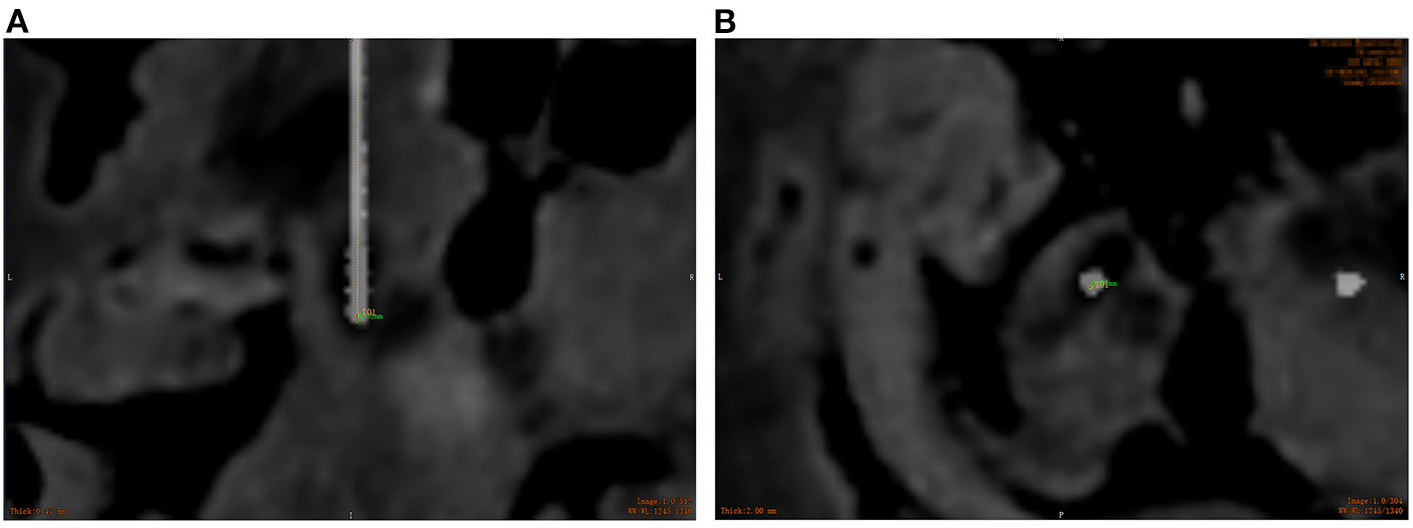
Overlay of the preoperative MR images with target trajectories and postoperative CT images to assess the electrode placement accuracy. **(A)** The radial error measured using the ruler tool; **(B)** the axial error measured using the ruler tool. (Orange represents the actual electrode position, and green represents the axial and radial error between the actual electrode and the preoperative plan).

**Figure 5:**
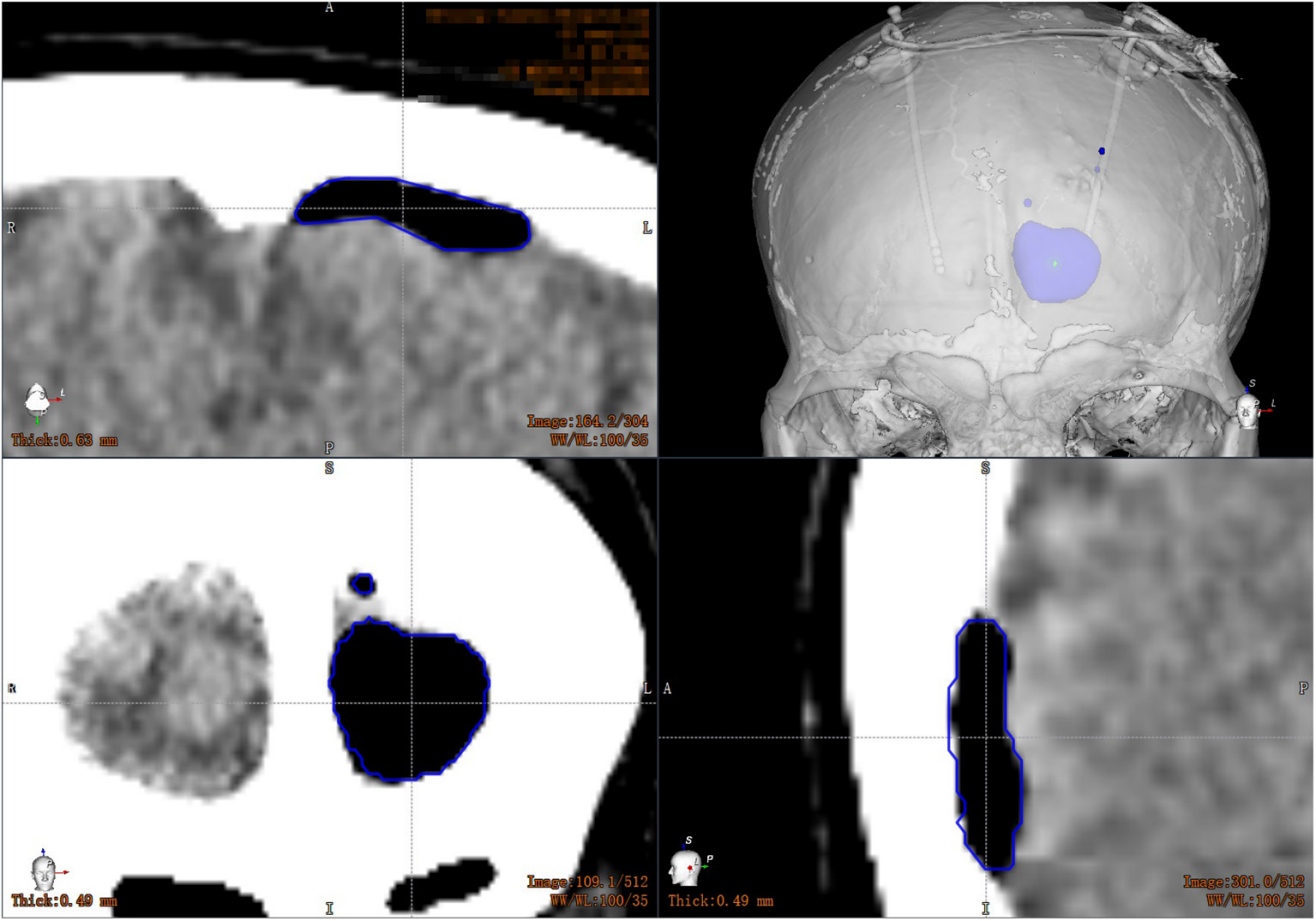
Volume calculation of intracranial air: the CT images are transmitted to the workstation, and the computer automatically calculates the volume of the patient's intracranial air layer by layer.

All patients underwent CT scan (slice thickness 0.625 mm) after operation. CT images were fused with the preoperative plan to assess the accuracy of electrode placement. The electrode accuracy was refered to the deviation between the actual contact center of the implanted electrode and the desired target point, which was evaluated using two measurement methods (Starr et al., 2010; VanSickle et al., 2019): the “radial error (RE)”, defined as the scalar distance measured from the view perpendicular to the planned trajectory, and the “axial error (AE)”, defined as the scalar distance along the planned trajectory measured from the view along the planned trajectory, as shown in Figure 4. In addition the volume of intracranial air (ICA) was calculated according to the addition of postoperative CT layer by layer measurements (Figure 5). All patients were followed up to confirm related complications.

### Statistical analysis

SPSS 23.0 software (IBM SPSS Statistics Inc., Chicago, IL, United States) was used for statistical analysis. The measurement data was represented by $\bar{x}\pm s$. The normality and homoschedasticity of the two groups of data were detected. If the variance was aligned, a one-way analysis of variance was performed, and if the variance was not uniform, the Wilcoxon test was performed. *P* < 0.05 was considered statistically significant.

## Results

**Figure 6:**
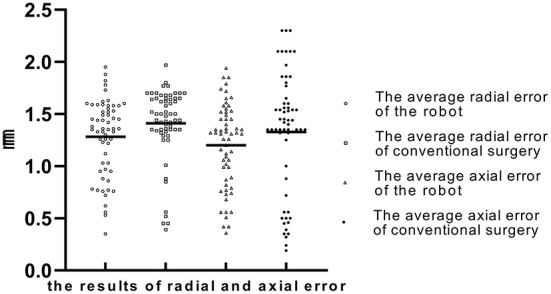
The average radial error and axial error of the robot group and the conventional surgery group (“◦” represents the average radial error of robot group; “□” represents the average radial error of conventional group; “Δ” represents the average axial error of robot group; “•” represents the average axial error of conventional group; The Y-axis is the error in millimeters).

**Figure 7:**
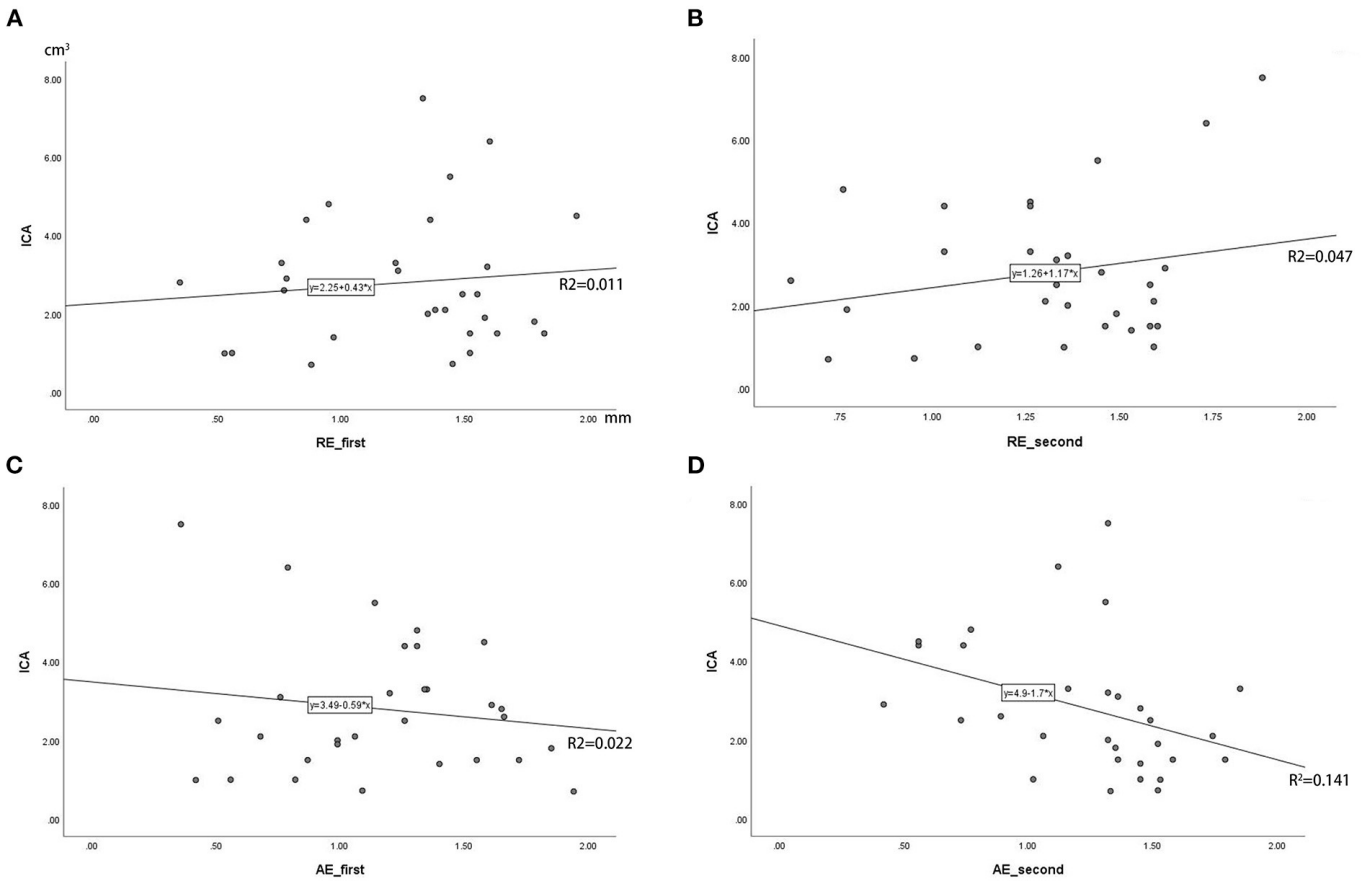
**(A–D)** Correlation analysis of intracranial air (ICA) volume with radial error and axial error (RE_first means the radial error of the electrode on the first implantation side, RE_second means the second implantation side; AE_first means the radial error of the electrode on the first implantation side, AE_second means the second implantation side; The Y-axis is the volume of ICA in cubic centimeters).

In the robot-assisted group, the average radial error was 1.28 ± 0.37 mm (0.35–1.95 mm), and the average axial error was 1.20 ± 0.40 mm (0.36–1.94 mm) (as shown in Table 2; Figure 6). The radial error of the electrode on the first implantation side is 1.25 ± 0.41, the second implantation side is 1.31 ± 0.31, and there is no statistical difference between the two sets of data (*P* = 0.54). The axial error of the electrode on the first implantation side is 1.17 ± 0.43, the second implantation side is 1.24 ± 0.38 and there is no statistical difference between the two sets of data (*P* = 0.52). The average time from drilling the burr hole to the completion of the second electrode implantation is 59.08 ± 13.48 min. The average volume of intracranial air in all patients was 2.80 ± 1.70 cm^3^, and there was no correlation between the intracranial air volume with radial errors or axial errors (Figure 7). In the group of conventional surgery, the statistical results show that the radial error and axial error of the stereotactic frame DBS operation are 1.41 ± 0.35 mm and 1.33 ± 0.58 mm, respectively, and there is no statistical difference between the robot assisted surgery and conventional surgery (*P*-values were *P* = 0.053 and 0.17, respectively). The volume of intracranial air on the postoperative CT of the patients with the stereotactic frame assisted electrode implantation was 11.86 ± 8.78 cm^3^, rang from 3.8 to 45.2 cm^3^.

**Table 2:** Comparison between the robot group and the conventional group.

	**Robot assisted surgery**	**Conventional surgery**	***p*-value**
RE	1.28 ± 0.37 mm	1.41 ± 0.35 mm	*P* > 0.05 (parametric test)
AE	1.20 ± 0.40 mm	1.33 ± 0.58 mm	*P* > 0.05 (non-parametric test)
Intracranial air	2.80 ± 1.70 cm3	11.86 ± 8.78 cm^3^	*P* < 0.05 (parametric test)

Twenty-six patients in the robot-assisted group received electrode implantation as planned, while the remaining patients adjusted the target after MER. In the conventional surgery group, six patients (6/30) adjusted target coordinates after MER. No deaths or associated hemorrhages, infections or poor incision healing occurred during hospitalization. In the robot-assisted group, 1 patients became indifferent after surgery, and 1 patient had short-term dyskinesia after surgery, but these recovered before discharge and did not cause lasting neurological deficits. In the group of conventional surgery, 2 patient developed dysarthria, 2 patients became indifferent after surgery, 2 patient had short-term dyskinesia after surgery, 1 patient developed electrolyte disturbances and 1 patient developed urinary tract infection. These also recovered before discharge.

## Discussion

DBS has become a mainstream surgical procedure over the last three decades (Harmsen et al., 2020). The positioning of the electrode is of utmost importance if an optimal clinical outcome, with minimal side-effects, is to be achieved (Hemm and Wårdell, 2010). Due to their compelling arguments of accuracy, steadiness and endurance, robotic systems have been used to assist in accurate electrode placement. Robotic systems have recently been used for DBS surgery because of the advantages they afford in maintaining excellent accuracy, simplifying workflow, and increasing operative efficiency (Goia et al., 2019; Ho et al., 2019; VanSickle et al., 2019). The mean radial target errors that frame-based and frameless robotic systems for DBS lead placement have reported were range from 0.6 to 1.40 mm (Lefranc and Le Gars, 2012; Neudorfer et al., 2018; Faraji et al., 2020). VanSickle et al. (2019) published a cohort study of 128 patients with a total of 241 lead implantations assisted by the Mazor Renaissance robot. In their study, the placement accuracy defined by the radial error for all final placements was 0.85 ± 0.38 mm, and the final cannula absolute depth errors along the planned trajectory were 0.64 ± 0.62 mm. Alice Goia et al. (2019) reported a cohort study of 24 patients, with a total of 44 lead implantations assisted by the ROSA robot. In their study, the 3D distance between the intended coordinates and the postoperative CT scan coordinates was 0.81 ± 0.51 mm right side and 1.12 ± 0.75 mm left side. Varma and Eldridge (2006) and von Langsdorff et al. (2015) both utilized the NeuroMate robot in their studies to assist the DBS electrode implantations. The mean (± SD) *in vivo* application accuracy was 0.86 ± 0.32 mm on a cohort of 17 patients with 30 basal ganglia targets, and the final electrode position varied from the planned trajectory by a mean of 1.7 mm, respectively.

In addition, Giridharan et al. (2022) used robotic stereotactic platform of ROSA for DBS surgery under general anesthesia, and used intraoperative fluoroscopic computed tomography for registration and postplacement verification. The robot used in our study can perform DBS surgery under local anesthesia, during which MER electrophysiological monitoring was used to assist in locating nuclear. The merits and demerits of the two methods are debatable. We describe our institution's result of the DBS surgery using the robotic system.

The accuracy of robot-assisted stereotaxy depends on several factors, including stable attachment of the robot to the skull and solid fiducial markers for registration (Neudorfer et al., 2018). The accuracy of the Remebot-assisted DBS procedure guided by a videometric tracker is comparable to that of other commercially available robots. Compared with the frame system, rembot system has some differences: the preoperative preparation of the robot is simplified and the cumbersome operation of coordinate adjustment is avoided; the robot can secondary registered in operation, simulate target correction, and reduce the human error of manual coordinate adjustment; the robot arm has a large operation range, 360° degree of freedom and automatic sensing device, and theoretically has no surgical blind area or surgical dead angle.

Previously, our team has used robots to perform stereotaxic surgery, including ventriculoperitoneal Shunting, ommaya reservoir implantation, biopsy, etc. (Liu et al., 2021a,b,c). These surgeries target relatively large areas, such as ventricles and tumors. But the size of the target of DBS, namely the nucleus, is relatively small, which makes robot-assisted DBS surgery need to emphasize stability and accuracy. Based on the previous experience, this study applied the robot to assist DBS surgery, to compare the accuracy of our streamlined robotic DBS workflow from robot assisted surgery and conventional surgery.

We calculated the electrode accuracy of 30 patients undergoing stereotactic frame DBS surgery in the same time period in this clinical center. The statistical results show that the radial error and axial error of the stereotactic frame DBS operation are 1.41 ± 0.35 mm and 1.33 ± 0.58 mm, respectively. In this study, the average radial error of robot assist group was 1.28 ± 0.37 mm (0.35–1.95 mm), and the average axial error was 1.20 ± 0.40 mm (0.36–1.94 mm). The accuracy of robot-assisted electrode implantation tends to be smaller than that of conventional stereoscopic frame surgery, but there is no statistical difference, which may require a larger sample size. It goes without saying that the use of robots can maintain the stability of the accuracy of electrode implantation. The accuracy of electrode implantation can effectively reduce the possibility of repeated adjustment of electrode position. Robotic placement therefore reduces the chance of human error and need for lead revision, which has been reported as high as 15.2–34.0% (Rolston et al., 2016). The complications caused by excessive adjustment of electrode position are mainly bleeding of cortex and basal ganglia. Repeated microlesion may also lead to decreased electrophysiological signals.

Postoperative electrode displacement is a research hotspot related to DBS surgery in recent years. The reasons for postoperative electrode displacement include postoperative ICA, mechanical errors in surgical methods, and errors caused by unskilled operators. Elias et al. (2007) found that the loss of cerebrospinal fluid during stereotactic surgery may cause the displacement of the cortex and deep brain structures. van den Munckhof et al. (2010) analyzed the errors of 26 DBS electrodes and found that the total displacement along electrode trajectories were 3.3 ± 2.5 mm. Besides, the ICA volume was (17 ± 24) cm^3^, and it is concluded that the displacement of the electrode on the X-axis, Y-axis and Z-axis are all related to the ICA volume. In this study, robot-assisted electrode implantation can be used for target planning before surgery, and accurately avoid important areas and cortical blood vessels. The traditional way to open the dura mater in DBS surgery is to cut the dura mater in a “cross” shape and then implant electrodes. Due to the large gap in opening the dura mater in this way, it often leads to more cerebrospinal fluid loss, causing brain tissue to shift and ICA, which affects the accuracy of electrode implantation. In this study, with the assistance of robots, the above problems were avoided: When planning the electrode implantation path before surgery, it is necessary to avoid cortical blood vessels, cerebral sulci and ventricles.

In this study, the average time from drilling the bone hole on the first side to the completion of the second electrode implantation is 59.08 ± 13.48 min. This also includes the time consuming of intraoperative microelectrode recording (MER). And postoperative CT showed that the volume of intracranial air was only 2.80 ± 1.70 cm^3^. Moreover, in this study, there is no correlation between the volume of postoperative intracranial air and the error (Figure 7). This means that the application of the robot can assist electrode implantation in a short time, smaller intracranial air volume does not affect the accuracy of intracranial electrodes, and its axial and radial errors are not significantly different from the figures in the literature. At the same time, the radial error of the electrode on the first implantation side is 1.25 ± 0.41, the second implantation side is 1.31 ± 0.31, and there is no statistical difference between the two sets of data, indicating that the use of robots can effectively avoid the impact of brain tissue displacement caused by the loss of cerebrospinal fluid during surgery.

Less postoperative ICA may reduce the incidence of postoperative psychiatric symptoms. Postoperative mental disorder in the robot-assisted group was 1 case, and 4 in the conventional group. The advantages of high precision of robot-assisted DBS enable the diameter of the dural incision to be controlled very small during the operation, which can slow down the rate of cerebrospinal fluid loss. Moreover, the target rarely needs to be adjusted during the operation, avoiding the potential changes in accuracy and safety caused by repeated position adjustment after cannula placement.

This study has some limitations. First, the number of patients with robot-assisted electrode implantation is small; Second, this study is not a randomized trial, and subsequent patients can be randomly assigned to implant electrodes assisted by a robot or a frame; Third, the problem of robot proficiency. The use of the robot needs to be run-in with the relevant personnel in the operating room and a long learning curve. In this study, there were errors in intraoperative cooperation. As the sample size increases and the use experience increases, there may be better results and trends. As for the limitations of the robot for DBS, for some tremor patients, the use of robot-assisted electrode implantation requires intraoperative general anesthesia, because the relative displacement of the robotic arm and the patient's body may occur with patient's symptoms, resulting in errors. In addition, general anesthesia surgery makes the patient unable to participate in the evaluation of intraoperative awake state, and anesthesia may affect electroacoustic signals.

## Conclusion

This study, which analyzed a cohort of robot-assisted electrode implantations guided by a videometric tracker in DBS procedures, demonstrates that the Remebot robotic system is accurate and safe. Moreover, the videometric registration workflow brings automation and ease that benefit both the patient and the neurosurgeon. Future work will provide a larger magnitude and the link between electrode placement and clinical efficacy.

## Data availability statement

The original contributions presented in the study are included in the article/supplementary material, further inquiries can be directed to the corresponding authors.

## Ethics statement

The studies involving human participants were reviewed and approved by the Ethics Committee of Beijing Tiantan Hospital. The patients/participants provided their written informed consent to participate in this study. Written informed consent was obtained from the individual(s) for the publication of any potentially identifiable images or data included in this article.

## Author contributions

F-ZM, J-GZ, and H-GL: conceptualization. F-ZM: data curation. F-ZM and D-FL: formal analysis and writing—original draft. D-FL, A-CY, KZ, J-GZ, and H-GL: methodology. A-CY, KZ, F-GM, J-GZ, and H-GL: supervision. A-CY: resources and project administration. F-GM: validation. J-GZ and H-GL: writing—review and editing. All authors contributed to the article and approved the submitted version.

## Funding

This work was supported by Beijing Municipal Science and Technology Commission (Grant Number: Z181100001618019).

## Conflict of interest

The authors declare that the research was conducted in the absence of any commercial or financial relationships that could be construed as a potential conflict of interest.

## Publisher's note

All claims expressed in this article are solely those of the authors and do not necessarily represent those of their affiliated organizations, or those of the publisher, the editors and the reviewers. Any product that may be evaluated in this article, or claim that may be made by its manufacturer, is not guaranteed or endorsed by the publisher.
